# Promoting the stability and adsorptive capacity of Fe_3_O_4_-embedded expanded graphite with an aminopropyltriethoxysilane–polydopamine coating for the removal of copper(ii) from water†

**DOI:** 10.1039/d1ra05160a

**Published:** 2021-11-03

**Authors:** Shunhui Wang, Wenjian Lao, Yi He, Heng Shi, Qihang Ye, Jing Ma

**Affiliations:** School of Chemistry and Chemical Engineering, Oil & Gas Field Applied Chemistry Key Laboratory of Sichuan Province, Southwest Petroleum University Chengdu 610500 China wangshunhui@swpu.edu.cn heyi@swpu.edu.cn +86 28 83037367; State Key Laboratory of Oil and Gas Reservoir Geology and Exploitation Chengdu Sichuan 610500 China; Southern California Coastal Water Research Project Authority Costa Mesa California 92626 USA

## Abstract

In this study, three magnetic graphites, namely, EGF, GAF, and GFA + KH550, were prepared, which were loaded either with Fe_3_O_4_ or with Fe_3_O_4_ and PDA or with Fe_3_O_4_, PDA, and KH550 onto expanded graphite. ATR-FTIR, XRD, XPS, SEM, TEM, and TGA characterization results showed that EGF, GAF, and GFA + KH550 were successfully prepared. Under the same initial copper concentration, the removal rates of copper ions by EGF, GFA, and GFA + KH550 were 86.2%, 96.9%, and 97.0%, respectively and the hazard index reductions of the three adsorbents were 2191 ± 71 (EGF), 1843 ± 68 (GFA), and 1664 ± 102 (GFA + KH550), respectively. Therefore GFA + KH550 exhibited better removal of Cu(ii) than EGF and GFA, for PDA and KH550 provided more adsorption-active sites like –OH and –NH. Here, the adsorption of GFA + KH550 fitted the pseudo-second-order kinetic and Langmuir models well within the testing range, which means that adsorption occurs on a monolayer surface between Cu(ii) and the adsorption sites. The intraparticle diffusion model and various thermodynamic parameters demonstrated that Cu(ii) was adsorbed on GFA + KH550 mainly *via* external surface diffusion and that the process was both endothermic and spontaneous. Recycling experiments show that GFA + KH550 has a satisfactory recyclability, and the way of direct recovery by magnets exhibits good magnetic induction. GFA + KH550 was applied in lake water and artificial seawater samples, and exhibited better removal of copper than that in DI water under the same environmental conditions for the existence of macromolecular organic matter. Furthermore, the adsorption capacity of copper ions was not relative to the salinity of water. The application of GFA + KH550 demonstrated the potential for application in water treatment procedures.

## Abstract 2

In this study, three magnetic graphites, namely, EGF, GAF, and GFA + KH550, were prepared, which were loaded either with Fe_3_O_4_ or with Fe_3_O_4_ and PDA or with Fe_3_O_4_, PDA, and KH550 onto expanded graphite.

## Graphical abstract

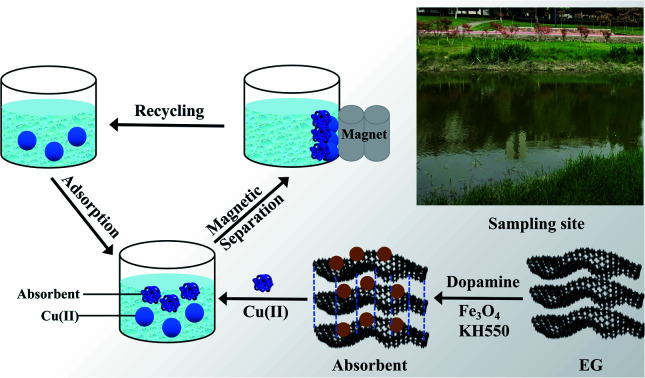

## 1. Introduction

Heavy metal pollution in water is a worldwide environmental issue and wastewater treatment presents a crucial measure for the mitigation of heavy metal contamination.^1^ Since heavy metals are non-degradable and toxic, their removal from water through the development of safe biomaterials is continuing to attract a great deal of attention.^2^ Various methods and techniques, such as chemical precipitation,^3^ redox,^4^ ion exchange,^5^ flocculation,^6^ adsorption, membrane filtration, electrodialysis, and photocatalysis^7,8^ have been developed to remove copper (Cu(ii)) from wastewater. Here, the adsorption method is regarded as the most promising for Cu(ii) removal, largely because of its operational simplicity, cost-effectiveness, and the possibility of regenerating the adsorbents.^9^ However, the recycling operation of current traditional adsorbents is difficult and applied in removing heavy metal from lakes and seawater.^10^ Therefore, a new and efficient method needs to be developed to improve adsorption performance in recycling and application in the actual environment. At the same time, magnetic materials for adsorption of heavy metals are commonly used for easily recycling.^11,12^

In fact, copper is known to pose a risk to aquatic ecosystems since it is generated by numerous industries and is toxic to the environment.^13^ Furthermore, although trace copper ions do not affect normal body functioning, an excessive intake of copper can lead to adverse effects on the immune system.^14^ Since copper contaminants are mainly discharged through vessel hull cleaning and maintenance processes,^15^ Cu element oxidizes to the more toxic Cu(ii) in highly saline water, which leads to intracellular ion imbalance and the inhibition of the enzymatic activity of carbonic anhydrase following excessive intake.^16^ The risk of Cu exposure through oyster consumption has also increased because of the copper contamination of seawater.^17^ Therefore, it is necessary to develop a suitable and easily treatment that can be applied for the removal of this metal from seawater.

Various carbon-based adsorbents have been developed for this purpose.^10,18,19^ Among them, expanded graphite (EG), a 3D worm-like and interconnected porous structure, has been identified as an excellent adsorbent for the removal of heavy metals from wastewater.^20–22^ EG has high solvent-accessible surface areas, excellent accessibility, and reactive functional groups that can be readily modified. In fact, when doped with magnetic components (*e.g.*, Fe_3_O_4_ and/or CoFe_2_O_4_), EG adsorbents can be conveniently isolated from the aqueous phase and subsequently regenerated and reused.^23,24^ However, since these magnetic particles do not readily disperse and adhere to the surface of graphene composites, the adsorbents exhibit short stability and reusability.^25^ Here, there is a strong likelihood that the immobilization of the Fe_3_O_4_ offers a solution to this issue. Various methods, including impregnation, co-precipitation, and calcination, have been employed to immobilize the magnetic nanocomposites on adsorbents.^26^ For example, Fe_3_O_4_ particles were wrapped in graphene oxide (GO) sheets *via* covalent bonding with –OH in GO and –NH_2_ in Fe_3_O_4_@SiO_2_–NH_2_ to produce recyclable magnetic adsorbents for removing Pb(ii),^27^ whereas a magnetic Fe_3_O_4_-encapsulated C_3_N_3_S_3_ polymer/rGO nanocomposite was synthesized and evaluated for the effective adsorption of Pb(ii) and Hg(ii) from aqueous solutions.^28^ However, these magnetic composites do not contain sufficient functional groups for adsorption.

PDA coatings are generally produced *via* the self-polymerization of dopamine on the surfaces of different materials. This procedure mimics the adhesive proteins secreted by mussels for attachment to various materials,^29^ due to inexpensive bio-inspired coatings utilized for the efficient adsorption of transition metal ions such as Cu(ii), Pb(ii), Ni(ii), and Zn(ii) from wastewater^30–32^ as well as oil–water separation/modification and biomedical treatment without the generation of secondary pollutants.^33–36^ Elsewhere, PDA has also been employed to stabilize the Fe_3_O_4_ attached on cellulose for its resorcinol adsorption from water.^37^

Meanwhile, silane coupling agents, such as 3-aminopropyltriethoxysilane, also known as KH550, has been used to coat Fe_3_O_4_/rGO composites to increase the electromagnetic wave absorption performance.^38^ Here, the KH550-modified composites had the capacity to enhance the removal efficiency in relation to various heavy metals, including Pb(ii) and U(iv).^39,40^ Furthermore, with the addition of KH550, covalent bonding can be formed with a broad range of organic and inorganic surfaces using PDA.^41^ However, although PDA and KH550 have been used separately to encapsulate Fe_3_O_4_ on a number of composites, no report exists in relation to EG/Fe_3_O_4_ (EGF) composites encapsulated with both PDA and KH550.

**Fig. 1:**
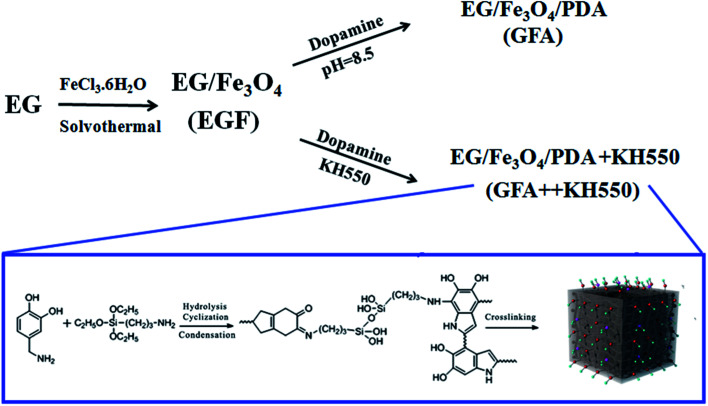
Synthesis of the PDA- and KH550-modified functional-group-modified rGO composites.

As such, the main aim of the present work was to prepare and characterized a inorganic–organic composite, *i.e.*, EG/Fe_3_O_4_/PDA + KH550 (GFA + KH550), for the adsorption and removal of Cu(ii) from water. This composite was prepared on a substrate of EGF functionalized by PDA and KH550. It was expected that the composite would provide numerous oxygen- and nitrogen-containing functional groups as active sites for binding metal ions *via* electrostatic interaction, coordination, hydrogen bonding, and π–π stacking interactions on both the basal and edge surfaces. To confirm the adsorption performance of the GFA + KH550, two adsorbents, EG/Fe_3_O_4_ (EGF) and EG/Fe_3_O_4_/PDA (GFA), were also compared. The performance of the composites was reflected in terms of the decrease in the hazard index (HI) of the Cu(ii) in deionized (DI) water, lake water, and artificial seawater. To better understand the adsorption mechanism, the macroscopic adsorption kinetics, isotherms, and thermodynamics of the adsorption were investigated, and the effects of various adsorption parameters, including contact time, initial concentration, pH value, and temperature, were also evaluated. The flowchart for the preparation of the three modified EGFs is shown in Fig. 1.

## 2. Materials and methods

### 2.1 Materials

EG powder (≥99%) was provided by Daying Juneng Technology and Development Co., Ltd. (Suining, China), dopamine hydrochloride and tri(carboxymethyl)aminomethane (≥98%) were purchased from Aladdin Industrial Corporation (Los Angeles, American), and γ-aminopropyl triethoxysilane (KH550 ≥ 90%), ethylene glycol (99.7%), anhydrous ethanol (99.8%), polyethylene glycol 2000 (AR), anhydrous sodium acetate (99%), ferric chloride hexahydrate (98%), standard Cu(ii) solution, hydrochloric acid (AR), sodium hydroxide (98%), and nitric acid (65–68%) were supplied by Chengdu Kelong Chemical Reagent Factory (Chengdu, China). The DI water used in the study was prepared using the molecular purifier produced by Shanghai Moller Scientific Instruments Co., Ltd (Molgy, Shanghai, China; pH = 6.5 ± 0.1).

### 2.2 Collection of water samples

The lake water was collected from Mengxi Lake of Southwest Petroleum University (N 30°49′55′′, E 104°10′58′′), with the pH of the lake water equal to 7.0 ± 0.1. Meanwhile, the artificial seawater was prepared by dissolving sea salts sourced from Guangzhou Aquarium Factory, China, in the lake water to achieve a salinity of 2.8‰, the water left to stand for around 24 h prior to use. The pH of the artificial seawater was 8.0 ± 0.05. The dissolved organic carbon (DOC) concentration (2.62 ± 0.019 mg L^−1^) in the lake water was quantified using Shimadzu's (Japan) Total Organic Carbon Analyzer (TOC-L) following filtration using a 0.45 μm cellulose membrane.

### 2.3 Preparation of polydopamine and KH550-modified EG materials

#### 2.3.1 Preparation of magnetic expanded graphite EG/Fe_3_O_4_ (EGF)

Samples of EG powder (1.5 g) and FeCl_3_·6H_2_O (1.5 g) were dispersed and dissolved in ethylene glycol (40 mL) *via* sonication at 25 °C for 0.5 h. After adding polyethylene glycol 2000 (1.0 g) and anhydrous sodium acetate (3.6 g), the mixture was stirred at 60 °C for 1 h before being transferred to a 100 mL Teflon-lined autoclave and heated at 200 °C for 12 h. After cooling, the mixture was centrifuged, and the final product was obtained through discarding the supernatant and washing the product using DI water (×3) and ethanol (×3). The EGF was then dried in a vacuum oven at 60 °C for 24 h.

#### 2.3.2 Preparation of composite EG/Fe_3_O_4_/PDA (GFA)

A 200 mg sample of the EGF was dispersed in DI water (50 mL) *via* sonication for 0.5 h. Following the addition of tris(carboxymethyl)aminomethane (60 mg) at 1.2 g L^−1^, the pH of the mixture was adjusted to 8.5 before the mixture was agitated for 0.5 h. Then, 100 mg of dopamine (2 g L^−1^) was added to the mixture, the mixture vigorously stirred for 24 h. The GFA was obtained through following the same protocols of centrifugation, washing, and drying as described above.

#### 2.3.3 Preparation of composite EG/Fe_3_O_4_/PDA + KH550 (GFA + KH550)

A 200 mg sample of the EGF was dispersed in DI water (50 mL) under ultrasonic conditions for 0.5 h. Following the addition of tri(carboxymethyl)aminomethane (60 mg) at 1.2 g L^−1^, the pH of the mixture was adjusted to 8.5 before being agitated for 0.5 h. Then, dopamine (100 mg) was added to the mixture, which was subsequently left for 4 h until the dispersion was complete. The silane coupling agent, KH550 (10 μL), was then added to the mixture, the mixture vigorously stirred for 24 h. The GFA + KH550 was obtained following the same protocols of centrifugation, washing, and drying as described above.

#### 2.3.4 Characterization

The synthesized composites were characterized using the following techniques: (1) Attenuated Total Reflection-Fourier Transform Infrared Spectroscopy (ATR-FTIR, 4000–500 cm^−1^, Bruker TENSOR27, German); (2) X-ray Diffraction Analysis (XRD, PANalytical, the Netherlands) using the X'Pert Pro diffractometer with a Cu Kα radiation source (scanning amplitude = 5°–80°, scanning rate = 2° min^−1^); (3) Thermogravimetric Analysis (TGA, TG209F1, Netzsch, Germany), performed under a N_2_ atmosphere in the range of 50–800 °C; (4) X-ray Photoelectron Spectroscopy (XPS, KRATOS, XSAM800, Britain), performed using an EscaLab 250 Xi X-ray photon–electron spectrometer with monochromatic Al Kα X-rays adopted as the excitation source and the C 1s peak used to check the energy scale; (5) Scanning Electron Microscopy (SEM); and (6) Transmission Electron Microscopy (TEM). The surface morphology and structure of the materials were observed *via* SEM (JSM-7500 F, JEOL, Tokyo, Japan) after being coated with 10 nm-thick Aurum, and subsequently *via* TEM (Tecnai G2F20, USA).

### 2.4 Evaluation of performance of the composite materials for removing Cu(ii) in aqueous solutions

#### 2.4.1 Comparison of the adsorbents

The adsorptive capacities of the adsorbents (EGF, GFA, and GFA + KH550) were evaluated by recording and comparing the Cu(ii) concentrations in a batch sorption experiment using spiked DI water conducted at 25 °C ± 1 °C. The DI water was spiked with a standard Cu(ii) solution to a Cu concentration of 15 mg L^−1^. After spiking and adjusting the pH value, the solution was thoroughly homogenized for 1 h using a stirrer (IKA C-MAG HS 7 control, German). The adsorbent (10 mg, 0.2 g L^−1^) was then added to the 50 mL Cu(ii) solution. Triplicates of the mixture were sonicated for 30 s prior to being placed in a water bath with a constant temperature (25 °C ± 1 °C), where they were shaken for 180 min. The Cu(ii) concentrations were measured in the water sub-samples that were taken at 5, 20, 30, 40, 50, 60, 90, 120, 150, and 180 min.

#### 2.4.2 Optimization of the adsorption conditions

After the best adsorbent was identified, *i.e.*, GFA + KH550, a series of experiments was conducted to optimize the adsorption conditions while altering one parameter at a time, which included pH (2, 4, 6, 7, 9, and 11, all at 25 °C ± 1 °C), temperature (25 °C, 35 °C, 45 °C, and 55 °C, all at pH = 7.0 ± 0.1), and initial concentrations (10, 15, 20, 25, 30, 40, and 50 mg L^−1^, all at 45 °C ± 1 °C and pH = 7.0 ± 0.1).

#### 2.4.3 Application of the GFA + KH550 in the lake water sample and the artificial seawater sample

The adsorptive capacities of the GFA + KH550 were used in the lake water and the artificial seawater samples spiked with Cu(ii), while it was also used in the spiked DI water sample as a control. The three types of water sample were doped with standard Cu(ii) to a concentration of 10 mg L^−1^ before being stirred for 1 h. All the adsorption experiments were conducted in triplicate at 45 °C ± 1 °C for 180 min.

#### 2.4.4 Regeneration tests

To assess the reusability of the GFA + KH550 in the DI water and the lake water sample, 10 mg of the GFA + KH550 was added to a 50 mL Cu(ii) (15 mg L^−1^) solution. The adsorption experiments were repeated for three cycles at 45.0 °C ± 0.5 °C and pH = 7.0 ± 0.1 for 180 min per cycle. At the end of each cycle, the adsorbent was regenerated *via* the application of an external magnetic field and through washing with 50 mL of HNO_3_ solution (0.1 mol L^−1^) and 50 mL of anhydrous ethanol followed by drying at 60 °C for 24 h. The Cu(ii) concentration was measured after 5, 10, and 20 cycles. Concurrently, the adsorption experiments were conducted using the control water, with the sampling time intervals the same as those used for the spiked water. The regeneration efficiency was expressed as a percentage, which was calculated by dividing the adsorption capacity following regeneration by the adsorption capacity before regeneration.

The triplicate water samples were filtered using 0.45 μm nylon-membrane syringe filters, the filtrates kept in 2% HNO_3_ before the Cu(ii) concentrations were measured *via* inductively coupled plasma spectrometer–optical emission spectrometry (Agilent ICP-OES 730, USA).

### 2.5 Data analysis

The removal of Cu(ii) from the aqueous solution by the adsorbents can be described in terms of the first-order kinetic model:

1 *C*_*t*_ = *b* + *a* × e^−*λt*^

where *C*_*t*_ is Cu(ii) concentration at time *t* (min) and *λ* (min^−1^) is the removal rate constant. The initial concentration *C*_0_ (mg L^−1^) is equal to (*a* + *b*) initial.^42,43^

The adsorption capacity of the adsorbents (*q*_*t*_, mg g^−1^) *versus* the time was calculated according to the corresponding concentration of Cu(ii), as described by eqn (2)2
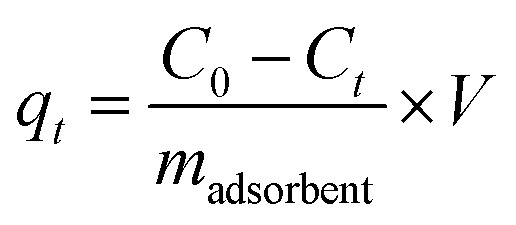
where *C*_0_ (mg L^−1^) is Cu(ii) concentration at the beginning of adsorption (*t*_0_), *V* (L) is the volume of the aqueous solution, and *m*_adsorbent_ (g) is the mass of the adsorbent. The adsorption capacity at equilibrium (*q*_e_, mg g^−1^) was calculated using eqn (2) with the equilibrium concentration *C*_e_ (mg L^−1^) instead of the *C*_*t*_.

The removal of Cu(ii) from the aqueous solution was assessed using the HI in terms of estimating the possible toxic effects on humans in health risk assessments. The HI was calculated using the following equation.^44,45^3
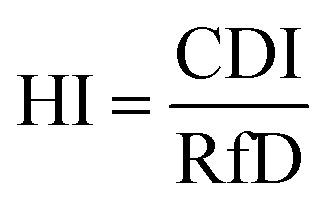
where RfD (the reference dose for Cu, 0.038 mg per kg per day)^46^ is a numerical estimate of the daily oral exposure to the human population (including sensitive subgroups such as children) that is unlikely to cause harmful effects during a lifetime, and CDI (mg per kg per day) is the chronic daily intake. The CDI was calculated as follows.^44^4
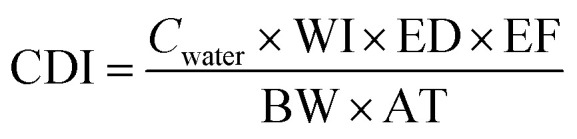
where *C*_water_ is Cu(ii) concentration in the water (mg L^−1^), WI is water intake rate (= 2 L per day), ED is the exposure duration (=30 years), EF is the exposure frequency (=350 days per year), BW is the body weight of an adult (=70 kg), and AT is the average time of exposure, which is 30 years for non-carcinogenic compounds or 70 years (lifetime) for carcinogenic compounds.

The adsorption kinetics was characterized using pseudo-first-order and pseudo-second-order kinetic models.^47^ The pseudo-second-order kinetic model is as follows:5
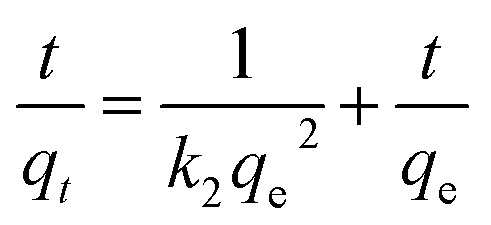
whereas the pseudo-first-order model is as follows:

6 Ln(*q*_e_ − *q*_*t*_) = ln *q*_e_ − *k*_1_*t*

where *k*_2_ (mg g^−1^ min^−1^) is the adsorption rate constant of the second-order rate model and *k*_1_ (min^−1^) is the adsorption rate constant of the first-order rate model.

To describe the diffusion-controlled processes in the adsorption stages, the intraparticle diffusion model was used as follows:

7 *q*_*t*_ = *k*_intra_(*t*)^1/2^ + *C*

where *k*_intra_ (mg g^−1^ min^−0.5^) is the intraparticle diffusion rate constant and *C* (mg g^−1^) is a constant related to the thickness of the boundary layer.^47^

Meanwhile, the Langmuir and Freundlich models were used to determine the maximum adsorption capacity of the adsorbent.^48,49^ The Langmuir and Freundlich models are as follows:8
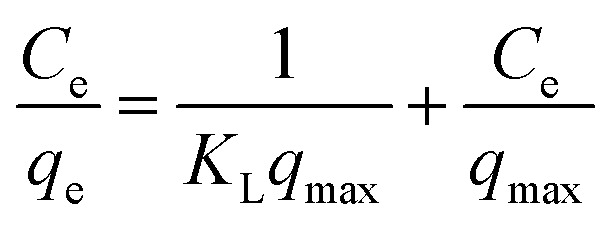
9
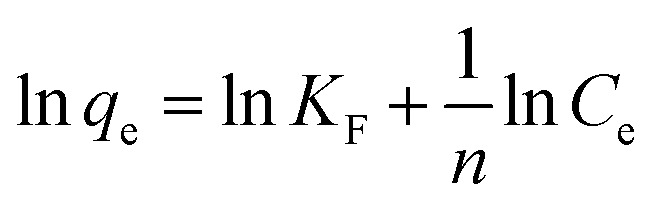
where *K*_L_ (L mg^−1^) is the Langmuir constant and *q*_max_ (mg g^−1^) represents the maximum adsorption capacity of the adsorbent. *K*_F_ is Freundlich constant representing adsorption capacity.

The thermodynamic parameters were used to determine the spontaneity, disorder, and endothermic or exothermic properties of the adsorption process. The standard enthalpy variable (*H*^*θ*^), standard entropy variable (*S*^*θ*^), and standard Gibbs free energy variable (*G*^*θ*^) relative to the adsorption process were calculated using the following equations:10
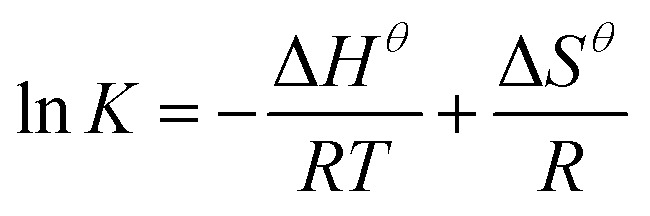

11 Δ*G*^*θ*^ = −*RT* ln *K*

where *R* is the gas constant 8.314 J mol^−1^ K^−1^ and *T* (K) is the temperature in kelvin; here, *K* (=*q*_e_/*C*_e_, L g^−1^) is the equilibrium constant at the different temperatures.

## 3. Results and discussion

### 3.1 Preparation strategy

The synthesis of the EGF, GFA, and GFA + KH550 is illustrated in Fig. 1. To synthesize the EGF, a co-precipitation method was used to load Fe_3_O_4_ particles on the EG. The Fe_3_O_4_ particles were immobilized on the EG *in situ* using FeCl_3_·6H_2_O *via* solvothermal reaction. The EGF was further coated with PDA, and PDA/KH550 to form GFA, and GFA + KH550, respectively.

It is worth noting that the bio-inspired modification of the EGF using the PDA and KH550 produced a large amount of hydroxyl and amino groups, which enhanced the hydrophilicity and adsorption performance. Dopamine was easily self-assembled and adhered to the surface of the material under mild conditions, which provided further hydrophilic functional groups *via* surface modification.^50^ The adherence of the KH550 to the composite also improved its hydrophilicity. The PDA- and KH550-modified composites could also be applied to other wastewater treatments owing to their ability to enhance the adsorption performance of methylene blue, and the separation of oil-in-water emulsion and dye with ultrahigh efficiency.^51–53^

### 3.2 Characterization of the composites

**Fig. 2:**
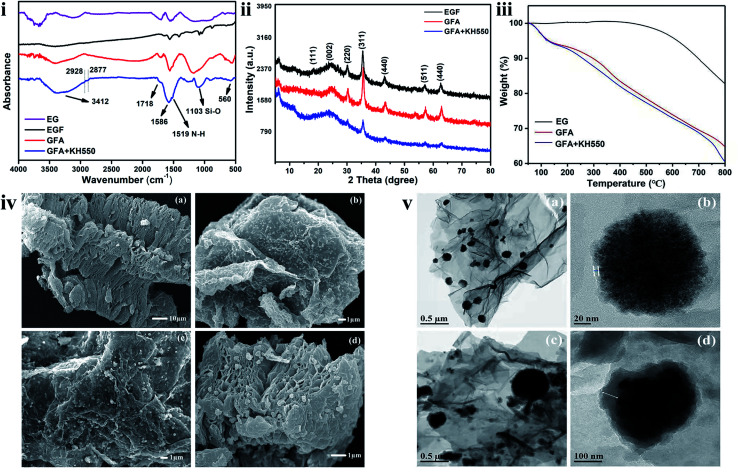
(i) ATR-FTIR spectra, (ii) XRD pattern, (iii) TGA curve, and (iv) SEM images of (a) EG, (b) EGF, (c) GFA, and (d) GFA + KH550, and (v) TEM images of (a and b) GFA and (c and d) GFA + KH550.

The materials were characterized *via* FTIR, XRD, TGA, XPS, SEM, and TEM analyses to evaluate the synthesis performance and structural features. The characteristic stretching frequencies of the EG, EGF, GFA, and GFA + KH550 in the FTIR spectra are shown in Fig. 2i. In each of these spectra, a broad absorption peak at around 3412 cm^−1^ was observed, which matched the characteristic peaks of –OH groups. With the addition of dopamine and KH550, the peak intensity of the GFA, and GFA + KH550 was significantly stronger than that of the EG and EGF, indicating that the dopamine and KH550 brought more –OH to form the GFA, and GFA + KH550. The bend vibration of –NH was observed at 1519 cm^−1^ in the GFA, and GFA + KH550 spectra. The absorption peaks observed at 2877 and 2928 cm^−1^ corresponded to the symmetric and non-symmetric stretching vibration of –CH_2_ afford by PDA and KH550, whereas the band at 1103 cm^−1^ resulted from the stretching vibration of the Si–O of the KH550. Meanwhile, the two peaks at 1718 and 1586 cm^−1^ were attributed to the characteristic peaks of the C=O and C=C of the EG. The existence of a strong peak of C=C stretching vibration overlapped with the C=N peak, and this likely led to the absence of C=N bending. Moreover, an absorption band at 560 cm^−1^ was observed, which was ascribed to the peak of Fe–O vibration. These results indicated that the dopamine and KH550 were coated on the EGF sheets and GFA, and GFA + KH550 were successfully synthesized.

The XRD patterns of EGF and the surface-modified EGF composites, including GFA, EGF + KH550, and GFA + KH550, are shown in Fig. 2ii. In all of the patterns, the diffraction peak at 2*θ* = 24.4° was attributed to the graphitic structure of EG and the modified EGF. Six characteristic peaks were observed for all the samples at 2*θ* = 18.3°, 30.1°, 35.5°, 43.0°, 57.1°, and 62.7°, which were attributed to the crystal planes of cubic spinel Fe_3_O_4_ of (111), (220), (311), (440), (511), and (440), respectively. Following the surface functionalization, the intensity of the GFA + KH550 diffraction peaks became weak owing to the coverage of the PDA and KH550 on the surface of the EGF. These results demonstrated that Fe_3_O_4_ nanoparticles were loaded on the EG. Moreover, the diffraction peak positions of the GFA + KH550 were consistent with those of the EGF, indicating the unchanged crystalline phase of Fe_3_O_4_ following the reaction.

Meanwhile, TGA was used to examine the modification content of the dopamine on the EG (Fig. 2iii). The weight of the pure EG did not change significantly up to 450 °C, indicating the high thermal stability of this material. With the increase in temperature, the EG functional groups gradually decomposed, whereas the carbon structure remained at the end of the reaction, with only around 17.0 wt% of oxygen functional groups left on the EG. The GFA and GFA + KH550 presented distinct degradation behavior compared with the EG. When the temperature was higher than 100 °C, the GFA and GFA + KH550 were clearly decomposed, with the final mass losses around 35.3 and 39.5 wt% following heating from 50 °C to 800 °C. The reason for the loss in the weight of the GFA related to the decomposition and detachment of the self-polymerized dopamine and Fe_3_O_4_ particles on the surface of the EG upon heating. Consequently, only 18.3 wt% of PDA and Fe_3_O_4_ remained on the EG. Similarly, on the EGF + KH550, the remaining amount of grafted KH550 following heating was around 4.2%.

The SEM images are presented in Fig. 2iv. The gap between the EG layers increased significantly following high-temperature treatment owing to the formation of a honeycomb-like porous structure. The surfaces of the EGF became rougher, and the distances between EG lamellae also increased after coating with the PDA + KH550 (Fig. 2ivd). The Fe_3_O_4_ nanoparticles generated in the subsequent solvothermal reaction process were uniformly loaded on the surfaces of the three materials. The binding between the Fe_3_O_4_ nanoparticles and the EF lamellae was assumed to be highly stable since the attachment of Fe_3_O_4_ nanoparticles did not diminish following the modification with the dopamine and KH550.

The TEM images further confirmed the successful loading of Fe_3_O_4_ nanoparticles on the EG substrate (Fig. 2v). Here, a thin coating layer on the surface of the Fe_3_O_4_ nanoparticles was observed (Fig. 2vb), indicating that the degree of self-polymerization of the dopamine on the surface of EGF was not high. However, after adding a small amount of KH550, a thicker organic layer formed because of the co-grafting of the dopamine and KH550 (Fig. 2vd), which indicated that the degree of copolymerization of the dopamine and KH550 was higher than that of the dopamine alone.

To further investigate the composition and elemental chemical states of the composite materials, XPS was employed. The same components, *i.e.*, C 1s (284.0 eV), O 1s (533.3 eV), Fe 2p_1_ (725.0 eV), and Fe 2p_3_ (711.9 eV), were appeared in the wide-scan spectra of the EGF (Fig. S1a†).^11,12,52^ The C 1s core-level spectrum of the EGF was divided into four peak components: C–C (284.6 eV), C–O (286.6 eV), C=O (288.4 eV), and O–C=O (290.3 eV) (Fig. S1b†).^11^ In terms of the GFA (Fig. S1c and d†), there was another characteristic peak at 401.3 eV, which was attributed to N 1s and the peaks of C–N at 285.4 eV in the attendant C 1s core-level spectrum.^11^ The Fe 2p_1_ and Fe 2p_3_ peaks of the GFA were clearly weaker than the peak of the EGF. Meanwhile, the peaks of N 1s, C–Si (282.4 eV)^52^ and C–N, as well as a Fe 2p intensity reduction, were also observed in the total spectrum and the C 1s spectrum of the GFA + KH550 (Fig. S1e and f†). The peaks at 168.35 and 89.76 eV corresponded to the Si 2s and Si 2p of the KH550 on the GFA + KH550.^12^ The presence of a C=N bond at 286.48 eV confirmed that there was a cross-linking reaction between the dopamine and the KH550, indicating that the two components had been successfully co-grafted (Fig. S1e†).^51^ The C 1s peaks of the EGF indicated that the EG contained numerous oxygen-bearing functional groups. Even after the loading of Fe_3_O_4_ particles, abundant active sites still remained (Fig. S1d†). The N 1s and C–N peaks shown in Fig. S1e and f† confirmed that the dopamine had successfully self-polymerized on the surface of the EGF (Fig. S1a, c and e†).

### 3.3 Capability of removing Cu(ii) from water and regeneration assessment

**Fig. 3:**
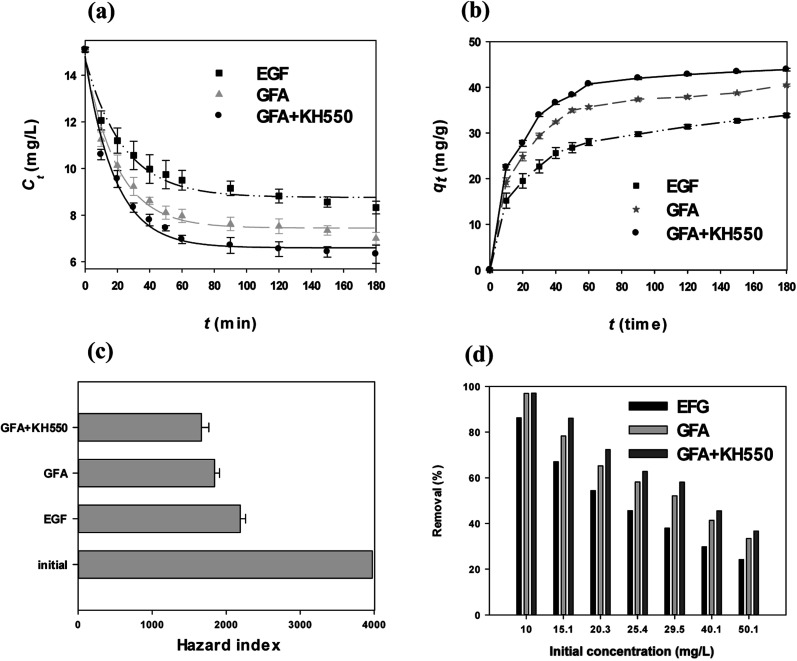
(a) Cu(ii) aqueous concentration *vs.* adsorption time of the four composites (EGF, GFA, EGF + KH550, and GFA + KH550), (b) concentration of Cu(ii) adsorbed on three composites (EGF, GFA, and GFA + KH550) *vs.* adsorption time, (c) the HI for non-carcinogenic health effects of Cu(ii) on adults before and after treatment using three composites (EGF, GFA, and GFA + KH550), and (d) Cu(ii) adsorption on the GFA + KH550 with different initial concentrations.

The removal of Cu(ii) by the three composites, *i.e.*, the EGF, GFA, and GFA + KH550, was observed; however, the extent of the removal differed among them (Fig. 3a). The Cu(ii) concentrations (*C*_*t*_) of the water treated with the EGF were significantly different from those in the water treated with the GFA and GFA + KH550, starting from 40 min until the end of the experiment at 180 min (one-way ANOVA, *p* < 0.05). The recorded *C*_*t*_s values were 8.33 ± 0.27 (EGF), 7.00 ± 0.26 (GFA), and 6.33 ± 0.39 mg L^−1^ (GFA + KH550), and the remove rate of copper ions the three adsorbents are 86.2%, 96.9%, and 97.0%. The GFA + KH550 demonstrated the highest efficiency in terms of Cu(ii) removal. The *C*_*t*_*versus* time (eqn (1)) fitted well (*R*^2^ = 0.9678 ∼ 0.9858) with the first-order kinetic model (eqn (12)–(14)):

12 *C*_*t*(EGF)_ = (8.76 ± 0.18) + (5.99 ± 0.35) × e^−(0.0425±0.0054)×*t*^

13 *C*_*t*(GFA)_ = (7.45 ± 0.15) + (7.39 ± 0.32) × e^−(0.0514±0.0048)×*t*^

14 *C*_*t*(GFA+KH550)_ = (6.59 ± 0.22) + (8.08 ± 0.47) × e^−(0.0508±0.0064)×*t*^

The adsorption capacity (*q*_*t*_, eqn (2)) of all three selected composites increased with the increase in adsorption time (Fig. 3b), with the *q*_*t*_ values approaching a plateau after 120 min. Meanwhile, the equilibrium adsorption capacities (*q*_e_, eqn (2)) were 33.9 ± 0.4 (EGF), 40.5 ± 0.2 (GFA), and 43.9 ± 0.3 mg g^−1^ (GFA + KH550). The *q*_e_ value of the GFA + KH550 was clearly the highest among the three composites. The PDA was combined with the KH550 *via* condensation to provide more adsorption-active sites.^12,54^ The IR, SEM, and TEM results were in line with these findings (Fig. 2).^52^

The HI was used to further illustrate the Cu(ii)-removal efficiency. For non-carcinogenic risk assessment, with the initial Cu(ii) concentration of 15.1 ± 0.1 mg L^−1^, the HI value of Cu for adults was 3974 ± 26, as calculated using eqn (3) and (4). The HI values shifted to 2191 ± 71 (EGF), 1843 ± 68 (GFA), and 1664 ± 102 (GFA + KH550) following the adsorption experiments, which lasted for 180 min. Compared with the initial Cu(ii) concentration, the HI risk of the Cu(ii)-spiked water treated using the GFA + KH550 declined more than twofold, with the HI reduction = 2310 ± 72, which was better than that of the EGF (1783 ± 75) and the GFA (2131 ± 105). For the initial concentration from 10 mg L^−1^ to 50.1 mg L^−1^, the removal efficiency of GFA + KH550 were 97.0–36.7%, which were the best for Cu(ii) among the three absorbents at the same initial concentration. This confirmed that the GFA + KH550 was the most effective in getting rid of Cu(ii) in water (Fig. 3d).

### 3.4 Adsorption kinetics and adsorption isotherms

**Fig. 4:**
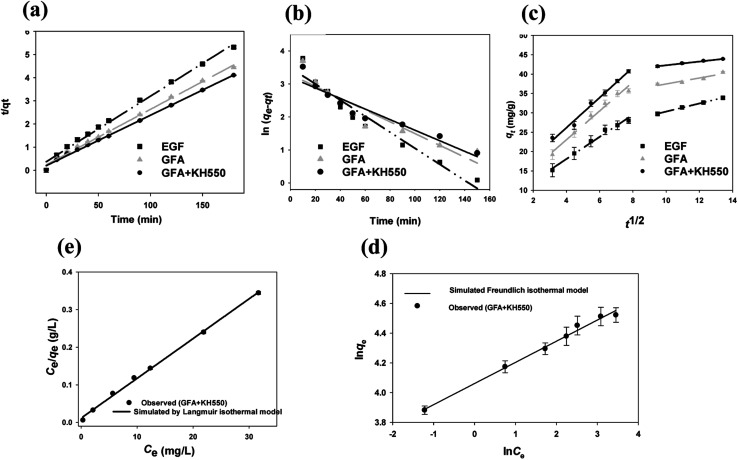
(a) Pseudo-second-order model fitting curves, (b) pseudo-first-order model fitting curves, (c) intraparticle diffusion model fitting curves (GFA + KH550 composites: 0.2 g L^−1^, pH = 7, 25 °C, initial concentration of Cu(ii) = 15.1 ± 0.1 mg L^−1^), (d) Langmuir isothermal fitting model, and, (e) Freundlich isothermal fitting model.

The Cu(ii) adsorption kinetics of the EGF, GFA, and GFA + KH550 were described well by the pseudo-second-order kinetic model (eqn (5), Fig. 4a). Here, the *R*^2^ values of the three composites were all above 0.99 (Table 1). Meanwhile, the equilibrium adsorption capacity (*q*_e2_) values were 35.3 (EGF), 41.7 (GFA), and 46.9 mg g^−1^ (GFA + KH550), which were very close to the experimental values (*q*_e,exp._ = 33.9, 40.5, and 43.9 mg g^−1^, respectively). In contrast, the *R*^2^ values obtained *via* the pseudo-first-order kinetic model (eqn (6)) ranged from 0.87 to 0.95 (Fig. 4b). The corresponding equilibrium adsorption capacity (*q*_e1_) values were 24.6 (EGF), 26.7 (GFA), and 34.7 mg g^−1^ (GFA + KH550). In line with previous studies, the pseudo-second-order kinetic model adequately described the measured Cu(ii) adsorption dynamic behavior.^54,55^

Kinetic parameters for the pseudo-first-order kinetic model, the pseudo-second-order kinetic model, and the intraparticle diffusion model of Cu(ii) adsorption on the GFA + KH550 (0.2 g L^−1^, pH = 7, 25 ± 1 °C, initial concentration of Cu(ii) = 15 mg L^−1^)

**Table:** 

Pseudo-first-order kinetic model	*q*_e1_ (mg g^−1^)	*q*_e,exp_ (mg g^−1^)	*k*_1_ (min^−1^)	*R* ^2^
EGF	24.6	33.9	0.0140	0.9108
GFA	26.7	40.5	0.0145	0.8678
GFA + KH550	34.7	43.9	0.0247	0.9469

**Table:** 

Pseudo-second-order kinetic model	*q*_e2_ (mg g^−1^)	*q*_e,exp_ (mg g^−1^)	*k*_2_ (mg g^−1^ min^−1^)	*R* ^2^
EGF	35.3	33.9	0.0021	0.9927
GFA	41.7	40.5	0.0024	0.9957
GFA + KH550	46.9	43.9	0.0024	0.9970

**Table:** 

Intraparticle diffusion model	*C*_1_	*k*_i1_	*R*_1_^2^
EGF	6.67 ± 1.04	2.85 ± 0.18	0.9850
GFA	8.17 ± 1.48	3.72 ± 0.25	0.9821
GFA + KH550	10.6 ± 1.2	3.89 ± 0.20	0.9897

**Table:** 

	*C*_2_	*k*_i2_	*R*_2_^2^
EGF	19.9 ± 0.3	1.04 ± 0.02	0.9991
GFA	29.7 ± 2.0	0.77 ± 0.17	0.9097
GFA + KH550	37.5 ± 0.2	0.48 ± 0.02	0.9963

**Table:** 

Pseudo-first-order kinetic model	*q*_e1_ (mg g^−1^)	*q*_e,exp_ (mg g^−1^)	*k*_1_ (min^−1^) (see ESI)	*R* ^2^
EGF	24.6	33.9	0.0140	0.9108
GFA	26.7	40.5	0.0145	0.8678
GFA + KH550	34.7	43.9	0.0247	0.9469

**Table:** 

Pseudo-second-order kinetic model	*q*_e2_ (mg g^−1^)	*q*_e,exp_ (mg g^−1^)	*k*_2_ (mg g^−1^ min^−1^)	*R* ^2^
EGF	35.3	33.9	0.0021	0.9927
GFA	41.7	40.5	0.0024	0.9957
GFA + KH550	46.9	43.9	0.0024	0.9970

**Table:** 

Intraparticle diffusion model	*C*_1_	*k*_i1_	*R*_1_^2^
EGF	6.67 ± 1.04	2.85 ± 0.18	0.9850
GFA	8.17 ± 1.48	3.72 ± 0.25	0.9821
GFA + KH550	10.6 ± 1.2	3.89 ± 0.20	0.9897

**Table:** 

	*C*_2_	*k*_i2_	*R*_2_^2^
EGF	19.9 ± 0.3	1.04 ± 0.02	0.9991
GFA	29.7 ± 2.0	0.77 ± 0.17	0.9097
GFA + KH550	37.5 ± 0.2	0.48 ± 0.02	0.9963

The adsorption processes of the three materials all exhibited two different stages, as identified by fitting the adsorption data in the intraparticle diffusion model (eqn (7), Fig. 4c). The diffusion rates of the first stage (*k*_i1_) were 2.85 ± 0.18, 3.72 ± 0.25, and 3.89 ± 0.20 mg g^−1^ min^−1/2^ for the EGF, GFA and GFA + KH550, respectively, whereas the diffusion rates of the second stage (*k*_i2_) were 1.04 ± 0.02, 0.77 ± 0.17, and 0.48 ± 0.02 mg g^−1^ min^−1/2^, respectively. Meanwhile, the intercept *C* values of the first stage were 6.67 ± 1.04 (EGF), 8.17 ± 1.48 (GFA), and 10.6 ± 1.2 mg g^−1^ (GFA + KH550), whereas those of the second stage were 19.9 ± 0.3 (EGF), 29.7 ± 2.0 (GFA), and 37.5 ± 0.2 mg g^−1^ (GFA + KH550).

The diffusion rate constant of the first stage was much higher than those of the second stage. For example, the *k*_i1_ of the GFA + KH550 was 8.1 times higher than the *k*_i2_. In contrast, the *C* values of the first stage were smaller than those of the second stage. The *C* value was related to the thickness of the boundary layer; *i.e.*, the larger the *C* value, the thicker the boundary layer. Therefore, the *C* values indicated that the thicknesses of the boundary layer of the GFA + KH550 were the highest among the four materials in both stages, which was in line with the SEM images. Meanwhile, the highest *q*_e2_ and *k*_i1_ of the first stage and the smallest *k*_i2_ of the second stage for the GFA + KH550 suggested that the first stage presented surface diffusion, whereas the second stage presented pore diffusion. The cross-linking between the PDA and the KH550 on the GFA + KH550 accelerated the first stage on the surface diffusion but slowed down the second stage on the pore diffusion.^56^ Therefore, the adsorption process of Cu(ii) on the GFA + KH550 was more significantly influenced by the external surface diffusion than the internal diffusion.

The Langmuir and Freundlich isotherm models are used to investigate the adsorption mechanism of Cu(ii) by GFA + KH550 absorbent material respectively. As shown in Fig. 4d and e, the Langmuir isotherm model shows better linear fitting than the Freundich isotherm model. Here, the maximum adsorption capacity (*Q*_max_) was 94.3 mg g^−1^, whereas *K*_L_ was 0.906 L mg^−1^, as based on the Langmuir isotherm model, while The *K*_F_ was 57.7 L g^−1^ calculated by eqn (9) based on the Freundich isotherm. The adsorption data for the GFA + KH550 fitted well with the Langmuir isotherm model (eqn (8), coefficient *R*^2^ = 0.9980) within the experimental range while the while the coefficient *R*^2^ of Freundich isotherm model was only 0.9743 (Fig. 4d). Accordingly, Langmuir model's coefficient is higher than Freundich model's, showing that Langmuir isotherm model better describes the adsorption process than Freundich model. Therefore, a monolayer adsorption occurred on the heterogeneous surface of EG + KH550 towards Cu(ii), and the adsorption mechanism was predominantly chemical adsorption.^57,58^ Surprisingly, as shown in Table 3, the *Q*_max_ of the GFA + KH550 was higher than those of multi-walled carbon nanotube-decorated Fe_3_O_4_ (Fe_3_O_4_/MWCNTs-COOH; 10.45 mg g^−1^), sodium dodecyl sulfate-coated Fe_3_O_4_ nanoparticles (SDS-Fe_3_O_4_ NPs; 24.2 mg g^−1^), oxidized sugarcane bagasse (SBox; 36.5 mg g^−1^), and oxidized cellulose (Cox; 76.8 mg g^−1^),^59–61^ and it was also higher than those (18.3–25.6 mg g^−1^) of several GO/Fe_3_O_4_ composites for the adsorption of Cu(ii).^62^ This was likely because there were more available adsorption sites on the GFA + KH550 than on the other materials.^61^

### 3.5 The effects of solution pH and temperature on adsorption

**Fig. 5:**
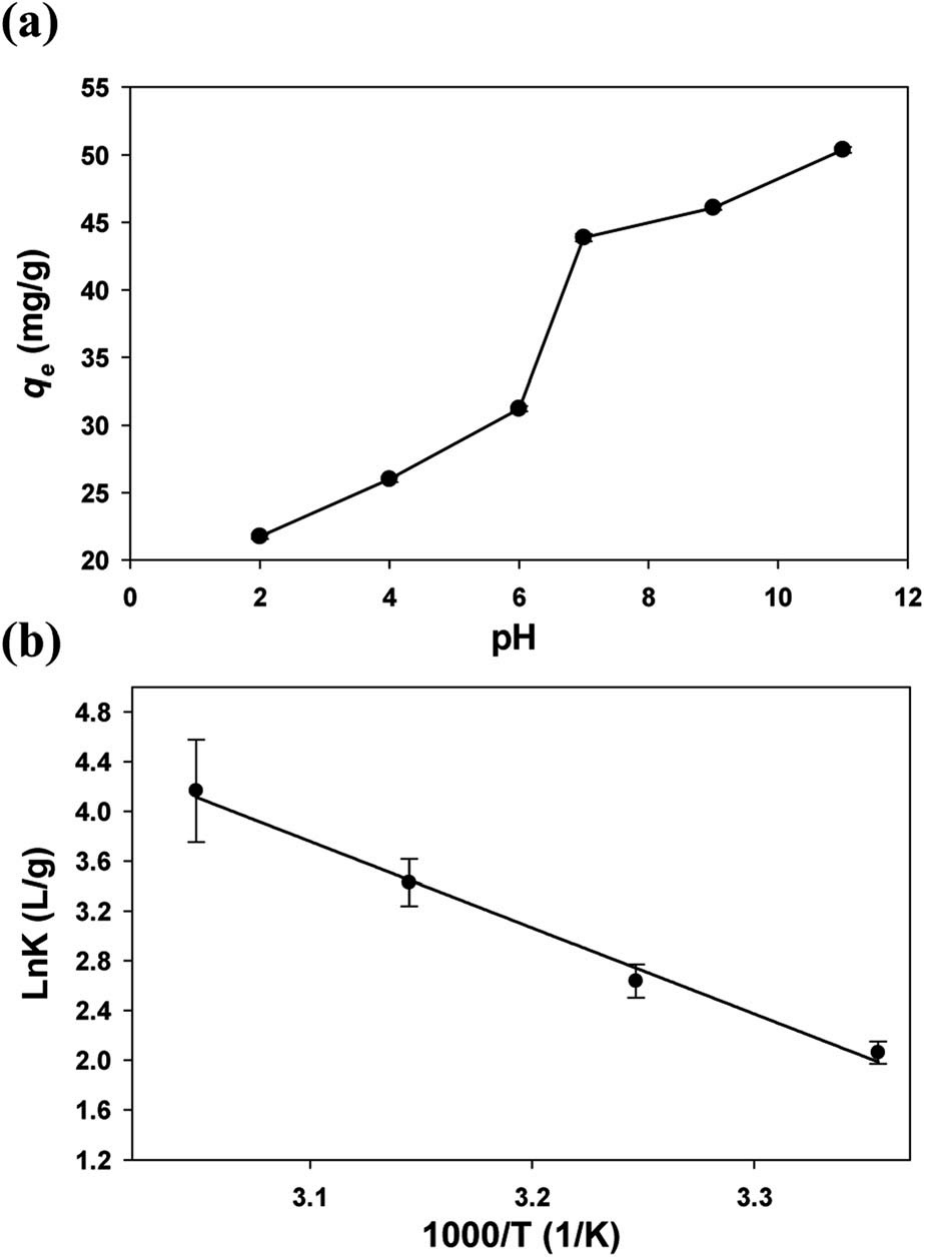
(a) Effect of pH on the Cu(ii) adsorption on the GFA + KH550 and (b) thermodynamic parameters for the adsorption of Cu(ii) on the GFA + KH550.

The solution pH had a significant impact on the adsorption performance. As shown in Fig. 5a, the *q*_e_ of the GFA + KH550 generally increased with the increase in pH value, with the values = 46.1 mg g^−1^ at pH 9 and 21.7 mg g^−1^ at pH 2 (eqn (2)). From pH 6 to pH 7, there was a pronounced increase in adsorption capacity. Non-electrostatic and electrostatic interactions are generally the main adsorption mechanisms that emerge on the surface of composite adsorbents.^63^ With the further increase of pH, Cu^2+^ existed in the form of Cu(OH)_2_ in water and was adsorbed by the GFA + KH550 easily.^64^ This correlation between *q*_e_ and pH value indicated that the adsorption was related to the interaction of the positive and negative charges on the surface of the adsorbent. The functional groups of the GFA + KH550 are protonated and take on the form of –NH_2_^+^ and –OH_2_^+^ under acidic conditions. The increase in solution pH leads to more primary amine and hydroxyl groups forming the structure of –NH and –OH, whereas Cu^2+^ and H^+^ always compete to occupy adsorption sites containing O and N.^12,52^ In addition, the surface of the GFA + KH550 is positively charged, which leads to electrostatic repulsion between the electropositive Cu(ii) and the sorbent's surface under acidic conditions. Here, the surface gradually becomes negatively charged when increasing the pH, and forces of attraction enhance the sorption by facilitating the interaction between the metal ions and the active sites.^65^ But the adsorption of Cu(ii) was discussed in the form of ions in this study. Therefore, we considered that pH = 7 was the most suitable values for Cu(ii) adsorption.

An increase in temperature was favorable to the adsorption. The relationship between thermodynamic equilibrium constant ln *K* and 1000/*T* was depicted by the van't Hoff plot with a high *R*^2^ value (0.9814, Fig. 5b). The Δ*H*^*θ*^ (16 295 ± 1579 J mol^−1^), Δ*S*^*θ*^ (70.8 ± 5.0 J [mol K]^−1^), and Δ*G*^*θ*^ values were all calculated (eqn (9) and (10), Table 2). The positive Δ*H*^*θ*^ values indicated that the adsorption presented an endothermic process. Therefore, increasing the temperature of the system would likely favor the adsorption.^12^ Meanwhile, the positive Δ*S*^*θ*^ value indicated an increase in randomness at the interface between the solid and the solution. Finally, the Δ*G*^*θ*^ value was negative, implying that the adsorption presented a spontaneous process.^66^

**Table:** The Cu(ii) adsorption thermodynamic parameters for the GFA + KH550

*T* (K)	Thermodynamic parameters			
	Ln *K* (L g^−1^)	Δ*G*^*θ*^ (J mol^−1^)	Δ*S*^*θ*^ (J [mol K]^−1^)	Δ*H*^*θ*^ (J mol^−1^)
298	1.94 ± 0.19	−4799 ± 143	70.8 ± 5.0	16 295 ± 1579
308	2.64 ± 0.13	−5488 ± 198		
318	3.43 ± 0.19	−6369 ± 159		
328	4.16 ± 0.41	−6859 ± 169		

**Table:** The maximum adsorption capacity (*Q*_max_)of different carbon-based material

Adsorbent	*Q*_max_ (mg g^−1^)	References
Multi-walled carbon nanotube-decorated Fe_3_O_4_ (Fe_3_O_4_/MWCNTs-COOH)	10.45	(Temnuch *et al.* 2020)
Sodium dodecyl sulfate-coated Fe_3_O_4_ nanoparticles (SDS-Fe_3_O_4_ NPs)	24.2	(Adeli *et al.* 2017)
Oxidized sugarcane bagasse (SBox)	36.5	(Rodrigues *et al.* 2020)
Oxidized cellulose (Cox)	76.8	(Rodrigues *et al.* 2020)
GO/Fe_3_O_4_	18.3–25.6	(Li *et al.* 2020)
GFA + KH550	94.3	This work

### 3.6 Application and regeneration of the GFA + KH550 in the field-collected water samples

**Fig. 6:**
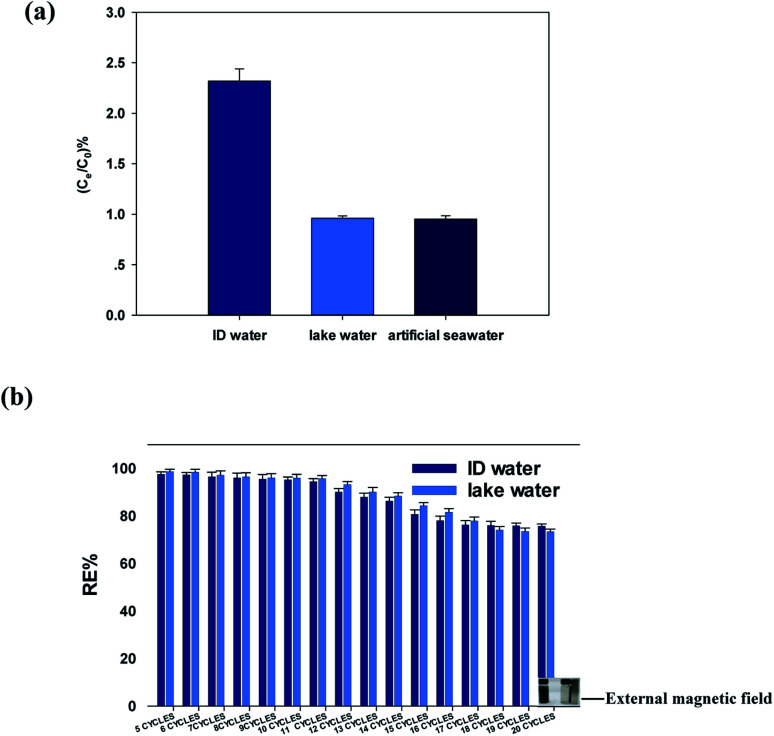
(a) Adsorption efficiencies of the GFA + KH550 in the spiked lake water, artificial seawater, and DI water samples; (b) regeneration efficiency (RE) of the GFA + KH550 in the spiked DI water and lake water samples.

The adsorption potential of the GFA + KH550 for Cu(ii) removal from the DI water, lake water, and artificial seawater samples was further examined. The ratios of Cu(ii) equilibrium concentration and initial concentration were 2.32% ± 0.12%, 0.960% ± 0.024%, and 0.952% ± 0.034%, for the DI water, lake water, and artificial seawater samples, respectively (Fig. 6a). Clearly, the ratio of Cu(ii) concentration in the DI water was significantly higher than those in the lake water (*p* < 0.05) and artificial water (*p* < 0.05), with the ratios similar for the latter two (*p* = 0.76). Using the same absorbent amount under the same environmental conditions, the adsorption capacity in the lake water and artificial seawater samples was slightly better than that in the DI water. This difference was likely due to the Cu(ii) associating with the DOC in the matrix of the field-collected water samples. It has previously been reported that macromolecular organic matters in natural water, such as humic acid and fulvic acid, provide specific adsorption sites that easily form complexes with metal ions in an aqueous system with a pH of 6–8.^67^ Here, although more inorganic ions exist in artificial seawater than in lake water, the adsorption capacity of Cu(ii) did not decrease, which indicated that the Cu(ii) adsorption was not affected by the salinity.

Meanwhile, regeneration was performed to investigate the adsorption potential of the GFA + KH550 in the DI water and lake water samples over multiple treatment cycles. Here, the regeneration efficiency in the first five cycles was 97.6% ± 0.5% and 98.7% ± 0.5% for the DI water and the lake water, respectively (Fig. 6b), whereas the regeneration efficiencies of the 10th cycle were 97.2% ± 0.5% and 97.0% ± 0.5%, respectively. Since there was no significant difference between the 5th and 10th cycles for the DI water (*p* = 0.383), the GFA + KH550 clearly exhibited good reusability after 10 cycles. However, the regeneration efficiencies of the 20th cycle were 75.7% ± 0.4% and 73.4% ± 0.4% for the DI water and the lake water, respectively, which indicated a clear decrease. This was likely because the Schiff base structure C=N formed by the condensation of PDA was broken and hydrolyzed by HNO_3_ during the regeneration process,^68,69^ with the breaking of the C=N bonds leading to the reduction in adsorption sites. Using other regeneration regents and the protection of the C=N bonds should be a focus of future investigations.

## 4. Conclusions

In this study, we demonstrated that the dual coating of PDA and KH550 significantly improves the adsorption capacity and reusability of EGF. The hybrid inorganic–organic composite, GFA + KH550, was easily prepared *via* solvothermal reaction. Based on the structural characterization, the GFA + KH550 exhibited excellent adsorption for Cu(ii), with its adsorption capacity affected by the time, pH, and temperature. The application of this material could reduce the HI of water contaminated with Cu(ii).

## Funding

This work was supported by the National Nature Science Foundation of China (51774245), Open Fund of Oil & Gas Field Applied Chemistry Key Laboratory of Sichuan Province (YQKF202008), the Opening Project of Oil & Gas Field Applied Chemistry Key Laboratory of Sichuan Province (YQKF202008) and Scientific Research Starting Project of Southwest Petroleum University (2019QHZ004).

## Author contributions

Shunhui Wang: data curation, writing – original draft, funding acquisition. Wenjian Lao: writing – review & editing, project administration. Yi He: supervision, funding acquisition. Heng Shi: methodology, investigation. Qihang Ye: investigation, validation. Jing Ma: methodology.

## Conflicts of interest

The authors have no conflicts of interest.

## Supplementary Material

RA-011-D1RA05160A-s001
