# Supplementary material for: Promoting the stability and adsorptive capacity of Fe3O4-embedded expanded graphite with an aminopropyltriethoxysilane–polydopamine coating for the removal of copper(ii) from water
Source: RSC Adv. 2021 Nov 3;11(56):35673–86. doi: 10.1039/d1ra05160a (PMC9043260; doi:10.1039/d1ra05160a)
Supplement: RA-011-D1RA05160A-s001 [file RA-011-D1RA05160A-s001.pdf]

Electronic Supplementary Material (ESI) for RSC Advances.
This journal is © The Royal Society of Chemistry 2021

Supplementary Data

Shunhui Wang,[a*] Wenjian Lao,[c] Yi He,[a, b*] Heng Shi,[a] Qihang Ye,[a] Jing Ma, [a]

[a] School of Chemistry and Chemical Engineering, Oil & Gas Field Applied Chemistry Key Laboratory of Sichuan Province, Southwest Petroleum University, Chengdu, China, 610500

[b] State Key Laboratory of Oil and Gas Reservoir Geology and Exploitation, Chengdu, Sichuan, China, 610500

[c] Southern California Coastal Water Research Project Authority, Costa Mesa, California, USA, 92626

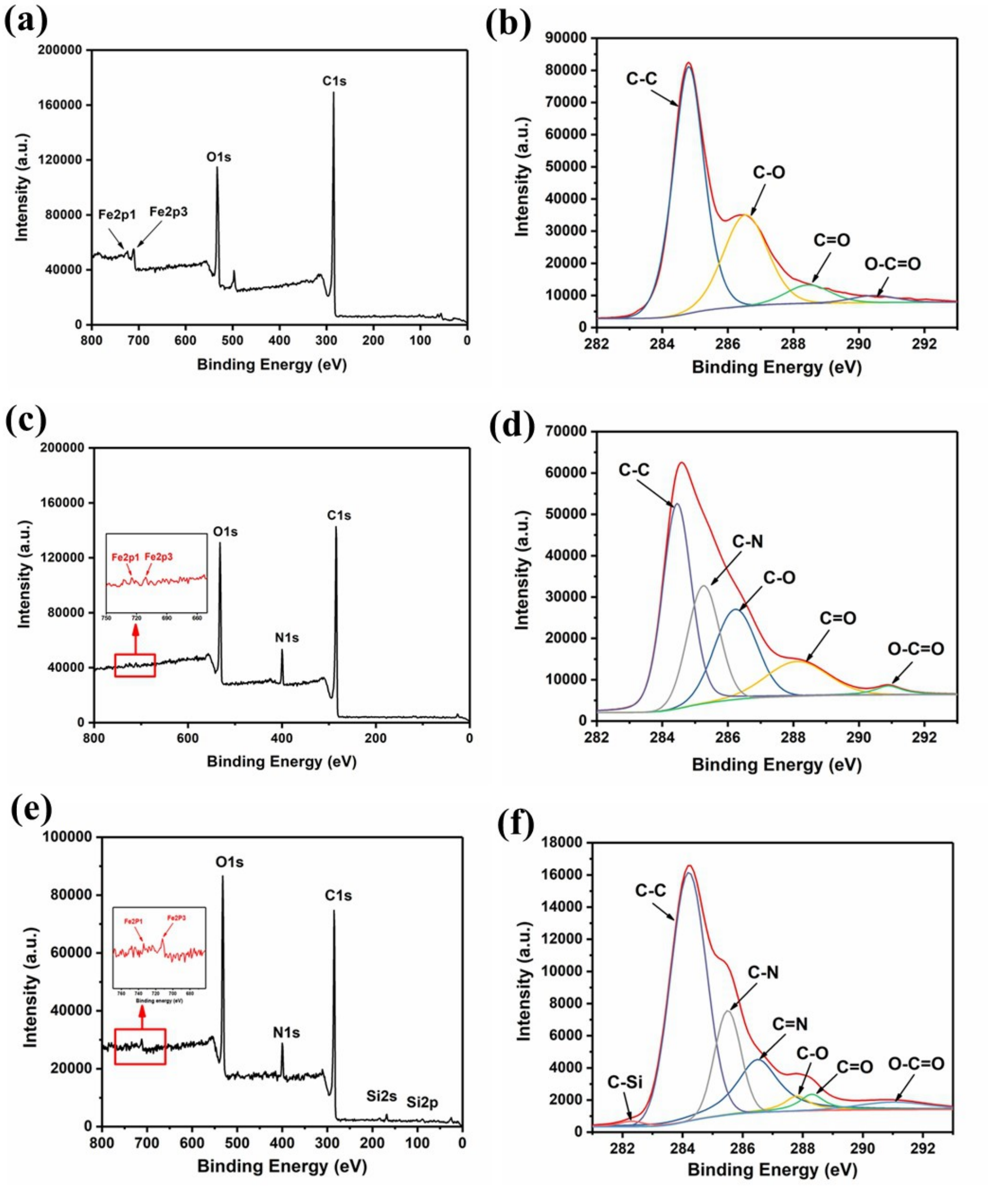

Figure S1 XPS wide scan and C1s core level spectra of (a and b) EGF, (c and d) GFA, (e and f) GFA+KH550
